# CSF‐1R inhibition disrupts the dialog between leukaemia cells and macrophages and delays leukaemia progression

**DOI:** 10.1111/jcmm.15916

**Published:** 2020-10-10

**Authors:** Kun Li, Wenfu Xu, Ke Lu, Yuxi Wen, Tianqing Xin, Yaqing Shen, Xueyan Lv, Shimin Hu, Runming Jin, Xiaoyan Wu

**Affiliations:** ^1^ Department of Pediatrics Union Hospital Tongji Medical College Huazhong University of Science and Technology Wuhan China; ^2^ Department of Hematopathology The University of Texas MD Anderson Cancer Center Houston TX USA

**Keywords:** acute lymphoblastic leukaemia, CSF‐1R, tumour‐associated macrophage, vincristine

## Abstract

Research in the last few years has revealed that leukaemic cells can remodel the bone marrow niche into a permissive environment favouring leukaemic stem cell expansion. Tumour‐associated macrophages (TAMs) are prominent components of the tumour microenvironment and play an important role in the onset and progression of solid tumours. However, little is known about their role in the development of acute lymphoblastic leukaemia (ALL). Using a unique mouse model of T‐ALL induced by injection of EL4 T‐cell lymphoma cells to syngeneic C57BL/6 mice, we report herein that ALL leads to the invasion of leukaemia‐associated monocyte‐derived cells (LAMs) into the bone marrow and spleen of T‐ALL mice. Furthermore, we found that leukaemia cells could polarize bone marrow–derived macrophages (BMDMs) into LAMs. In turn, LAMs were able to protect leukaemia cells from drug‐induced apoptosis in vitro. Therapies targeted against the TAMs by inhibiting colony stimulating factor‐1 receptor (CSF‐1R) have emerged as a promising approach for cancer treatment. In this study, we demonstrate that CSF‐1R inhibition inhibits the viability of BMDMs, blocks LAMs polarization and reduces the abundance of LAMs in T‐ALL mice. In vivo, combination treatment of CSF‐1R inhibitor and vincristine (VCR) dramatically increased the survival of T‐ALL mice and delayed leukaemia progression compared with VCR monotherapy. Finally, these data reinforce the role of microenvironments in leukaemia and suggest that macrophages are a potential target for the development of novel therapeutic strategies in T‐ALL.

## 1 INTRODUCTION

Acute lymphoblastic leukaemia (ALL) is the most common paediatric malignancy, of which T‐cell ALL (T‐ALL) accounts for 10%‐15% of total cases. Despite the excellent response to chemotherapy, relapse can occur in approximately 20%‐25% of paediatric T‐ALL patients. Resistance to therapy and relapse often indicate a poor outcome.^1^, ^2^ Therefore, investigation of novel strategies for T‐ALL therapy has important clinical significance.

Macrophages are heterogeneous cell populations originating from monocytes. They exist in almost all tissues and play crucial roles in the maintenance of tissue homeostasis.^3^, ^4^, ^5^ In response to different stimuli, macrophages have great plasticity and can differentiate into several functional states. Typically, macrophages can be divided into two major subsets, M1 and M2.^6^ M1 macrophages refers to classically activated macrophages by cytokines such as interferon γ, tumour necrosis factor‐α (TNF‐α) or lipopolysaccharides (LPSs). The M1 macrophages are involved in the host defence against different pathogens and play a role in anti‐tumour immunity by producing numerous inflammatory mediators, such as interleukin 6, reactive oxygen species and nitric oxide.^7^ In contrast, M2 macrophages, known as alternatively activated macrophages, are stimulated by interleukin‐4 (IL‐4) or interleukin 13 and are associated with anti‐inflammatory responses. They secrete transforming growth factor‐β, interleukin 10 (IL‐10), arginase and other cytokines that cause immune suppression, angiogenesis and tissue repair.^8^

However, tumour‐associated macrophages (TAMs) are a subset of macrophages present in the microenvironment of solid tumours.^9^, ^10^ They show distinctive transcriptional profiles and characteristics from either typical M1 or M2 macrophages. Generally, TAMs are considered as predominantly M2‐like and have pro‐survival effects including enhanced angiogenesis, tumour cell invasion, migration and inhibition of anti‐tumour responses.^9^, ^10^, ^11^

Despite a good understanding of the role of macrophages in solid tumours, little is known about this in haematopoietic malignancies. Compared with solid tumours, leukaemia and leukaemia‐associated monocyte‐derived cells (LAMs) have different clinical features because of their special microenvironment.^12^ Leukaemic stem cells can remodel the bone marrow (BM) niche into a self‐reinforcing leukaemic niche.^12^, ^13^, ^14^ This might enhance the leukaemic stem cells’ quiescence, leading to tumour progression and chemotherapy resistance. Recently, it was shown in mouse models that acute myeloid leukaemia (AML) cells can polarize macrophages towards a leukaemia‐supporting state in a growth factor independence 1–dependent manner, suggesting that AML‐associated macrophages play an important role in the progression of AML.^15^ However, the role of macrophages in ALL development and progression in vivo is not fully defined.

Colony stimulating factor‐1 receptor (CSF‐1R) is mainly expressed by monocytes/macrophages and myeloid‐derived suppressor cells.^16^ The CSF‐1/CSF‐1R signal pathway is important in the regulation of recruitment, differentiation and functions of macrophages.^17^, ^18^ Recent studies have shown that CSF‐1R blockage by inhibitors or antibodies improves the efficacy of chemotherapy in multiple solid tumours, such as glioma, cervical and mammary tumour.^17^, ^18^ Furthermore, targeting CSF‐1R has been proven effective on haematopoietic malignancies, such as multiple myeloma, chronic lymphocytic leukaemia (CLL) and a subset of AML.^19^, ^20^, ^21^, ^22^, ^23^

Based on these findings, we hypothesized that CSF‐1R blockage could be an important novel approach to improve ALL therapy. Here, we employed T‐ALL mice to identify a specific population of LAMs in vivo. Using a highly selective CSF‐1R inhibitor, BLZ945, we found that the pharmacologic inhibition of CSF‐1R decreased the abundance of LAMs in BM and spleen of leukaemia mice. Moreover, BLZ945 had a synergistic effect with vincristine (VCR) and prolonged survival of T‐ALL mice. Our data suggest that CSF‐1R small‐molecule inhibitors may be novel strategies for T‐ALL therapy.

## 2 MATERIALS AND METHODS

### 2.1 Animals

C57BL/6 mice were purchased from the Animal Centre of the Institute of Tongji Medical College, Huazhong University of Science and Technology, Wuhan, China. Female mice aged 6 to 8 weeks were used and maintained in the specific pathogen‐free certified animal facility in the same centre. Mice were randomly assigned to experimental groups. The experiment strictly abided by the ethical guidelines and approval of the Animal Care and Use Committee of Tongji Medical College, Huazhong University of Science and Technology.

### 2.2 Cell line

EL4 cells, a mouse malignant T‐cell lymphoma cells line, was purchased from Boster Biological Technology Co., Ltd.(Wuhan, China) and maintained in DMEM (Gibco, Thermo Fisher Scientific, USA), supplemented with 10% foetal bovine serum (FBS) and 1% penicillin/streptomycin.

### 2.3 Mouse models

Female C57BL/6 mice age 6‐8 weeks were injected through the tail vein with 2 × 10^6^ lymphoblastic EL4 cells. The mice were killed 7 days after the first injection, and peripheral blood (PB), BM and spleen was collected for further analysis. After confirmation of leukaemia cell engraftment, the mice were divided into 3 groups with 8 mice per group: group 1: control group mice injected with PBS; group 2: mice injected with 150 μg/kg VCR intraperitoneally; and group 3: mice injected with VCR and BLZ945 (20 mg/kg) (Apexbio, USA) intraperitoneally. The treatments were administrated every 3 days for total 5 times. The mice were killed at the indicated time points. BM and spleen were collected, and a single‐cell suspension was prepared for fluorescence‐activated cell sorting analysis. Briefly, BM cells were obtained by flushing femurs and tibiae and suspended in cold PBS + 2% FBS. Spleen cells also were harvested as a single‐cell<> suspension in the same buffer. Cells were filtered through a 70‐μm cell strainer to remove cell clumps, and red blood cells were removed using Red Blood Cell Lysis Buffer. Then, cells were resuspended in PBS containing 0.5% bull serum albumin, stained with a combination of antibodies, washed and subjected to cell sorting analysis.

### 2.4 Generation of macrophages

Primary macrophages were derived from mice BM cells and cultured for 7 days in DMEM‐containing 10% FBS, 20 ng/mL recombinant murine M‐CSF (Peprotech, 315‐02, USA) and 1% penicillin/streptomycin. Formation of mature bone marrow–derived macrophages (BMDMs) was evaluated using flow cytometry analysis and fluorophore‐conjugated antibodies to detect cells expressing CD11b and F4/80.

### 2.5 Flow cytometry analysis

The cells were analysed by cytoFLEX flow cytometer (Beckman Coulter, USA), using the FlowJo software. The antibodies used in present study were FITC anti‐mouse/human CD11B Antibody (Biolegend, Cat. No. 101205, USA), APC anti‐mouse F4/80 Antibody (Biolegend, Cat. No. 123116, USA), APC anti‐mouse CD206 (MMR) Antibody (Biolegend, Cat. No. 141707, USA) and PE anti‐mouse Ly6G Monoclonal Antibody (Elabscience, Cat. No. E‐AB‐F1108D, China).

### 2.6 Apoptosis assay

BMDMs were seeded in a 6‐well plate and treated with 50, 100 or 1000 nmol/L BLZ945 for 48 hours. Then, cells were harvested and stained with fluorescein isothiocyanate (FITC)–conjugated Annexin‐V and propidium iodide by using the Annexin‐V‐FITC staining kit (BD, USA). After co‐cultured with different macrophage‐conditioned media with or without BLZ945, EL4 cells were treated with VCR (75 ng/mL) or vehicle and subjected to apoptosis analysis. After staining, cells were analysed in cytoFLEX flow cytometer. The percentages of cells undergoing apoptosis were analysed by FlowJo Software.

### 2.7 Real‐time PCR analysis

Total RNA was extracted using RNAiso Plus reagent (Takara, Japan) and reverse transcribed with a PrimeScript RT reagent kit for RT‐PCR (Takara, Japan), following the manufacturers’ protocols. RT‐PCR was performed using an ABI Prism 7500 Sequence Detector (Applied Biosystems, Foster City, CA). The expression level of target genes was analysed by the relative quantity (RQ) value calculated using the ΔΔCt method. Experiments were performed in triplicates for each sample. Table S1 provides details regarding the primers used in our study.

### 2.8 Haematoxylin and eosin (H&E) staining

The histopathological analysis was performed using H&E staining. Briefly, after being fixed in 10% formalin, the liver or spleen was dehydrated and embedded in paraffin. Then, the paraffin‐embedded sample was sliced into 4 μm sections, and tissue sections were stained with H&E.

### 2.9 Immunohistochemistry (IHC) staining

IHC was performed on 4 μm thick formalin‐fixed, paraffin‐embedded tissue sections mounted on glass slides. Paraffin sections were stained with anti‐mouse CD68 antibodies (Abcam, Ab955, USA), then incubated overnight at 4°C in a humidified chamber. Next, the sections were washed with PBS three times and then incubated with secondary anti‐mouse horseradish peroxidase–conjugated antibody for 20 minutes at room temperature. Stained slides were photographed using an OLYMPUS BX53 biological microscope and analysed by Image‐pro plus 6.0 (IPP 6.0) software.

### 2.10 Enzyme‐linked immunosorbent assay

Macrophages were cultured in the presence or absence of EL4 conditional media (EL4‐CM) for 48 hours. IL‐10 production in macrophage supernatants or in serum of mice was measured by the commercially available enzyme‐linked immunosorbent assay kits according to the manufacturer's instructions (Elabscience, Wuhan, China).

### 2.11 Statistical analysis

Data represent mean ± SEM of representative experiments. Comparisons between two groups were analysed by unpaired Student's t test, whereas comparisons among multiple groups were analysed by one‐way ANOVA. Survival analysis was performed using Kaplan‐Meier statistics. Analyses were done using GraphPad Prism 8.0 (GraphPad Software, CA). *P* < 0.05 was considered statistically significant.

## 3 RESULTS

### 3.1 LAMs accumulated in the BM and spleen of T‐ALL mice

**Figure 1:**
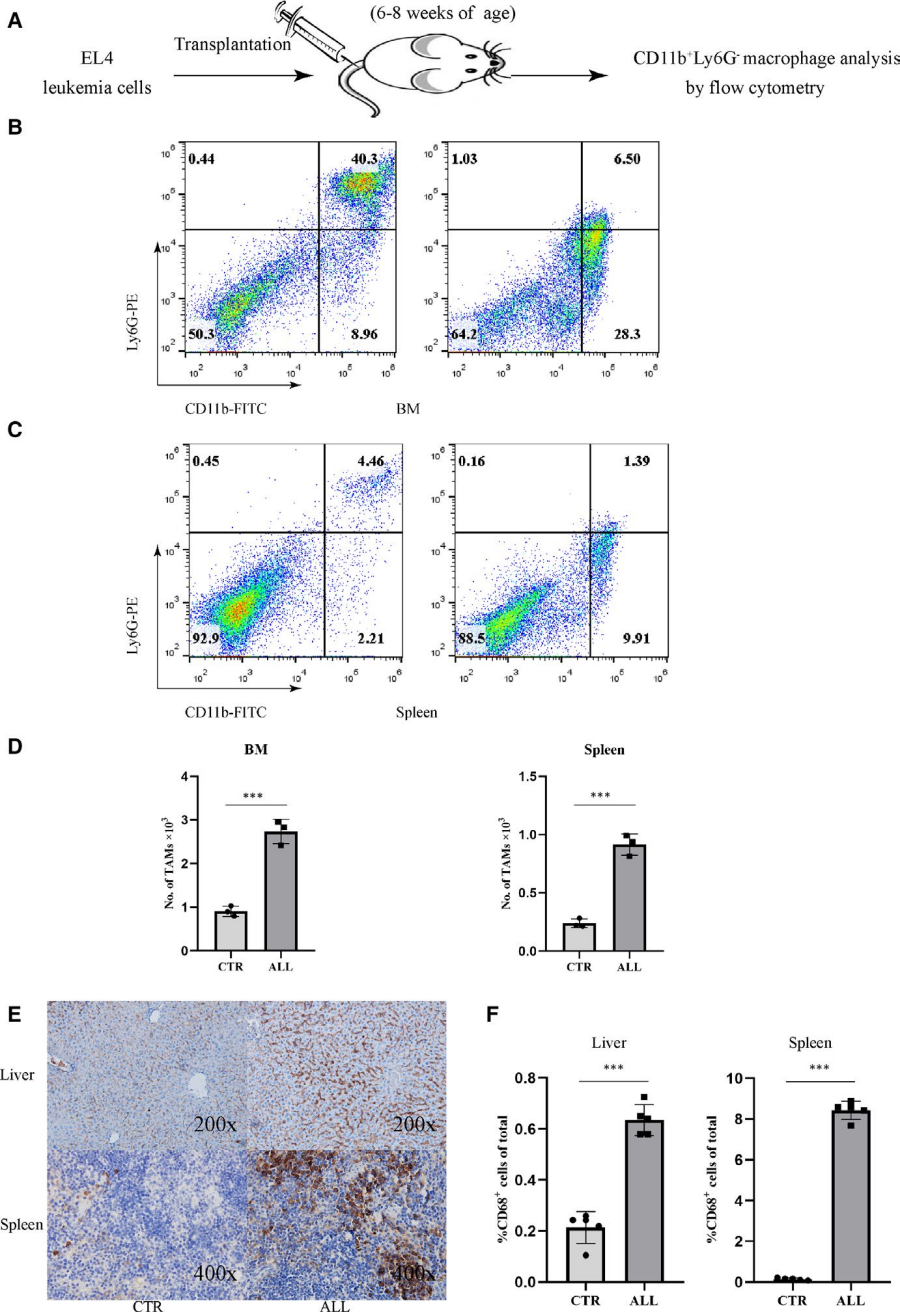
LAMs accumulate in the BM and spleen of T‐ALL mice. A, Schematic illustration of the experimental design. Approximately 2 × 10^6^ EL4 cells were transplanted into C57BL/6 mice. When the mice developed T‐ALL at 7 d after EL4 transplantation, CD11b^+^Ly6G^−^ macrophages were analysed by flow cytometry. B, The ratio of CD11b^+^Ly6G^−^ macrophages in the BM cells derived from mice transplanted with EL4 cells (right panel), compared to control mice (left panel). C, Ratio of CD11b^+^Ly6G^−^ macrophages in the spleen derived from mice transplanted with EL4 cells (right panel), compared to control mice (left panel). D, Bar graph shows the absolute numbers of CD11b^+^Ly6G^−^ macrophages in the BM (left, n = 3) or spleen (right, n = 3) of leukaemic mice, compared to control (out of 10^4^ cells acquired by FACS). E, Liver and spleen sections of control (left) or leukaemia mice (right) were stained using an anti‐CD68 antibody to identify macrophages (brown staining). F, Frequency of CD68^+^ macrophages in the liver (left, n = 5) or spleen (right, n = 5) of control or leukaemia mice

To investigate the role of macrophages in the development of T‐ALL, murine T lymphoblastic EL4 cells were transplanted into immunocompetent mice (Figure 1A and Figure S1). By using a similar gating strategy for studying LAMs in AML as reported by Yahya et al,^15^ we determined the percentage of LAMs defined as CD11b^+^Ly6G^−^ in the BM and spleen of T‐ALL mice. The frequency of CD11b^+^Ly6G^−^ LAMs in the BM and spleen of T‐ALL mice was significantly higher than in normal mice (Figure 1B‐D). We then quantitatively analysed macrophage content in liver and spleen tissue in T‐ALL mice by immunohistochemistry using an anti‐CD68 antibody, a marker of macrophages, as reported by Kong et al,^24^ The ratio of CD68^+^ macrophages in T‐ALL mice significantly increased when compared with mice receiving vehicle (Figure 1E,F).

### 3.2 Leukaemic cells polarized macrophages into LAMs

**Figure 2:**
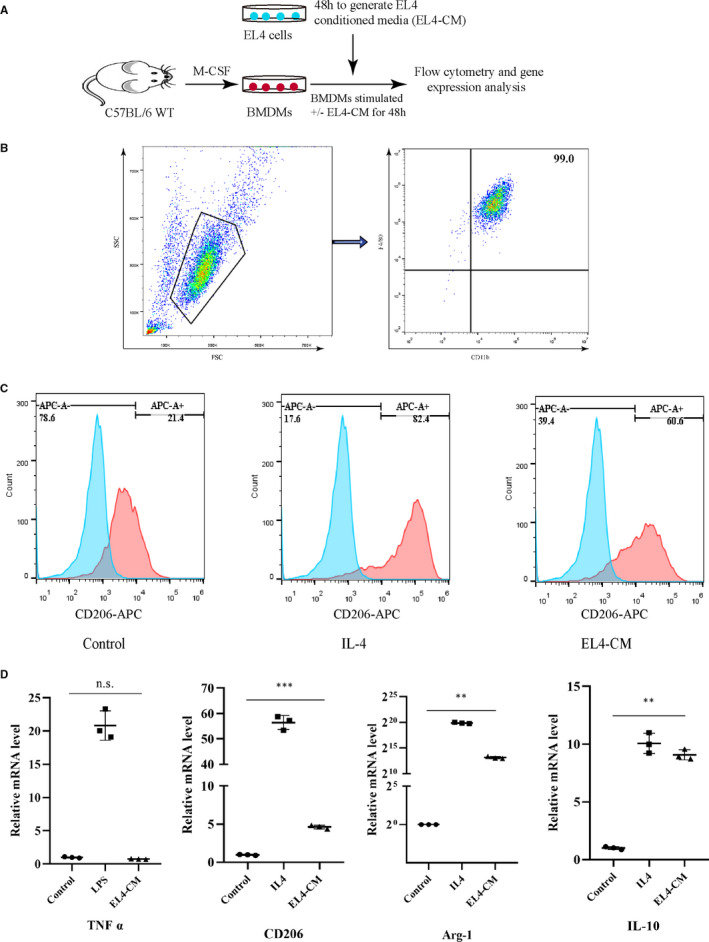
Leukaemic cells polarize macrophages into LAMs. A, Scheme of co‐culture assay of EL4 cells and BMDMs. B, Mature BMDMs were defined as CD11b^+^F4/80^+^ subpopulations. C, CD206^+^ BMDMs was analysed by flow cytometry after IL4 (20 ng/mL) or EL4‐CM stimulation for 48 h. D, BMDMs were stimulated with LPS (100 ng/mL) or IL‐4 (20 ng/mL) or EL4‐CM for 48 h and M1‐ or M2‐associated genes were characterized by RT‐PCR, n = 3

Macrophages are characterized by specific gene expression patterns, cytokine secretion and cell surface molecules which can be affected by the tumour microenvironment. To assess the ability of EL4 cells to educate macrophages and affect their polarization, BMDMs were co‐cultured with EL4‐CM to evaluate the expression of M1/M2 macrophage hallmarks (Figure 2A). Simultaneously, BMDMs were also cultured in the presence of either LPS (100 ng/mL), a M1 stimulator, or IL‐4 (20 ng/mL), a M2 stimulator. First, we successfully induced mature BMDMs identified by CD11b^+^F4/80^+^, a typical marker for BM macrophage^25^ on day 7 with 99.0% purity (Figure 2B). To confirm that leukaemic cells can polarize macrophages to LAMs, we first studied the expression of CD206 by flow cytometry analysis as CD206 is a M2 macrophage‐specific marker and plays an important role in tumour cell proliferation. As depicted in the Figure 2C, the percentage of CD206^+^ cells was significantly higher in co‐culture than in culture alone. Then, we further investigated the mRNA levels in co‐cultured BMDMs. We found that the expression of CD206, Arg1 and IL‐10 mRNA, characteristic for M2 macrophages, was significantly increased in co‐culture when compared with BMDMs culture alone. In contrast, there was no significant difference with regard to the expression of TNF‐α between control and EL4‐CM group, which is characteristic of M1 macrophages (Figure 2D).

### 3.3 BLZ945 inhibited the viability of BMDMs and blocked LAMs polarization

**Figure 3:**
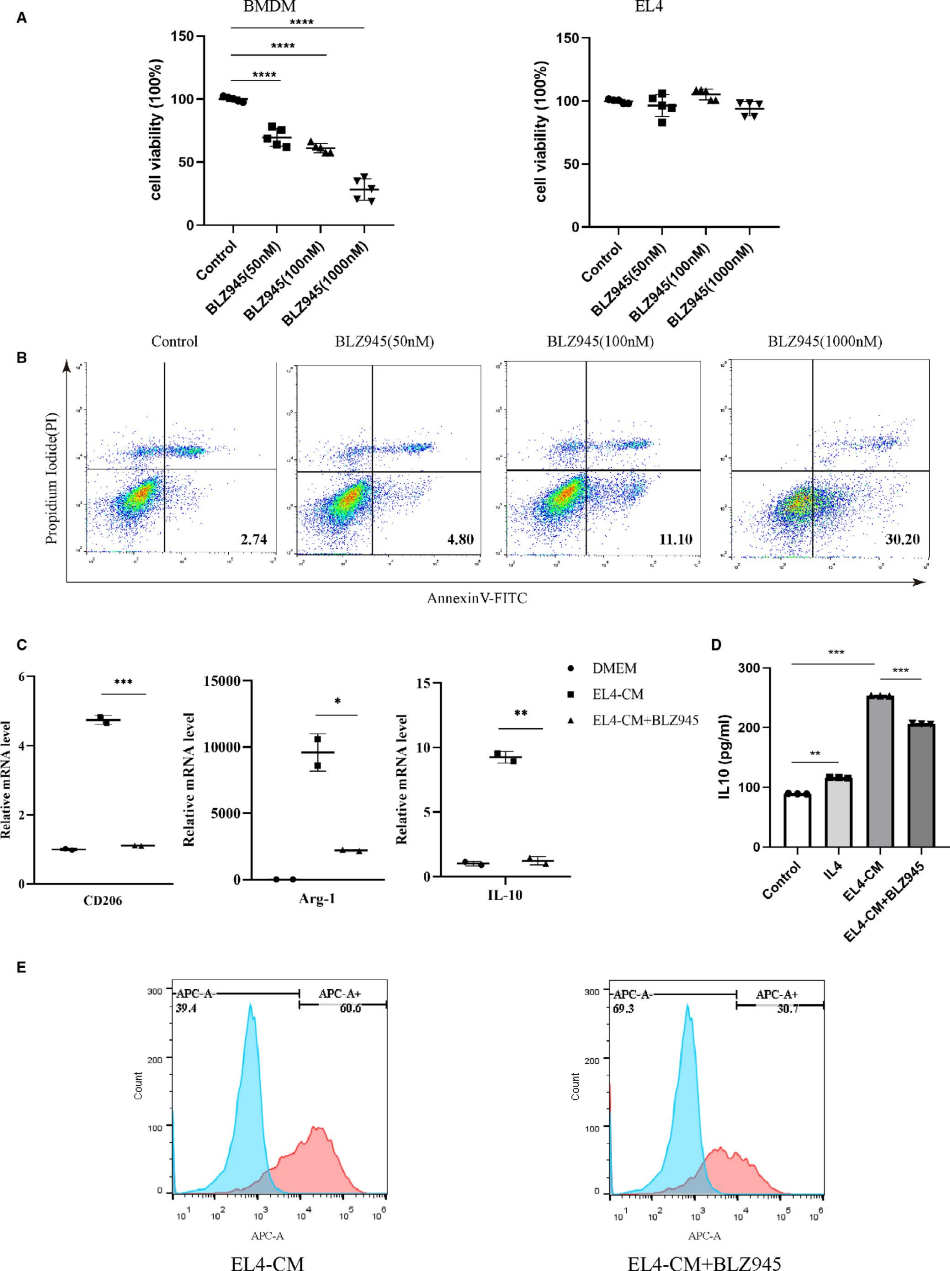
BLZ945 inhibits the viability of BMDMs and block LAMs polarization. A, Cell viability of BMDMs or EL4 cells after treated with different concentrations of BLZ945 for 48 h (n = 5). B, Apoptosis of BMDMs after treated with different concentrations of BLZ945 for 48 h. Apoptotic BMDMs were detected by double staining with Annexin‐V and propidium iodide and measured by flow cytometry. C, Graph showing expression of genes in BMDMs when stimulated with EL4‐CM in the presence or absence of BLZ945 (n = 3). D, Level of IL‐10 in supernatants of BMDMs treated with EL4‐CM for 48 h in the absence or presence of BLZ945 (n = 3). BMDMs were stimulated with IL‐4 (20 ng/mL) for 48 h as a positive control. (E) CD206^+^ BMDMs was analysed by flow cytometry after EL4‐CM stimulation for 48 h in the presence or absence of BLZ945

BLZ945 is a CSF‐1R inhibitor that inhibits the proliferation of TAMs. Therefore, we evaluated the inhibitory efficiency of BLZ945 in BMDMs and EL4 cells. First, BLZ945 treatment significantly inhibited the viability of macrophages in a dose‐dependent manner and induced apoptosis in BMDMs (Figure 3A,B). Importantly, BLZ945 treatment had no direct effects on the proliferation of lymphoblastic EL4 cells (Figure 3A). What's more, BLZ945 treatment also significantly induced apoptosis in BMDMs in the presence of EL4‐CM for 48 hours. (Figure S2). To explore how CSF‐1R inhibition regulates macrophage polarization, we first co‐cultured BMDMs and EL4 cells with or without BLZ945 for 48 hours. Exposed to EL4‐CM significantly increased mRNA levels of CD206, Arg‐1 and IL‐10, as determined by RT‐PCR, and that was blocked in the presence of BLZ945 (Figure 3C). The production of IL‐10, which is characteristic of TAMs, was significantly increased in EL4‐CM‐treated BMDMs and also abrogated by BLZ945 (Figure 3D). We next examined CD206, a well‐established M2 marker, in co‐cultures and found that CD206 also decreased in response to BLZ945 (Figure 3E). Together, this suggests that BLZ945 inhibits the viability of BMDMs in vitro and that, after CSF‐1R inhibition, EL4‐treated BMDMs lose LAMs polarization.

### 3.4 Signalling between macrophages and EL4 cells was abrogated by CSF‐1R inhibition

**Figure 4:**
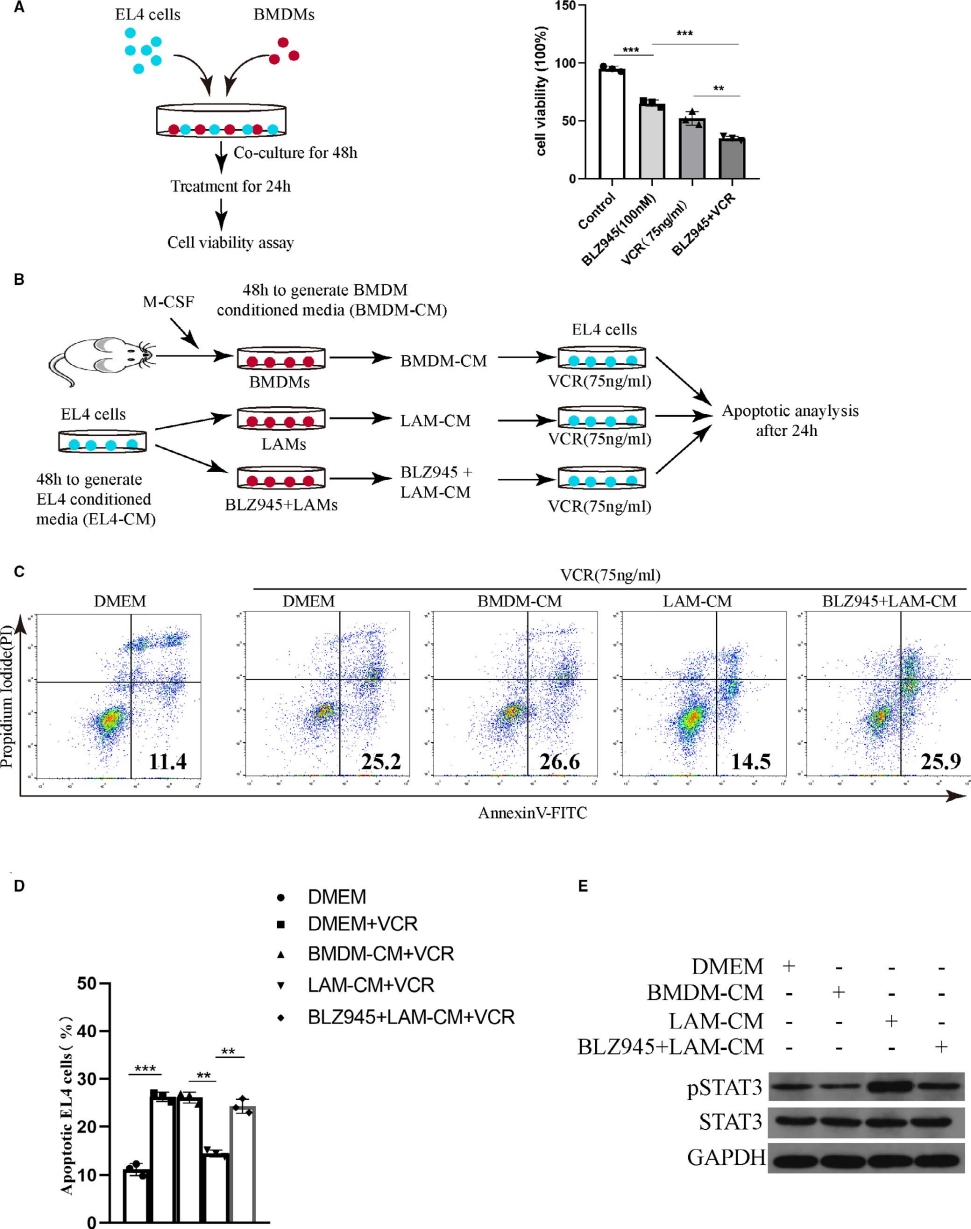
Signalling between macrophages and EL4 cells is abrogated by CSF‐1R inhibition. A, Scheme of co‐culture assay of EL4 cells and BMDMs (left panel). Co‐cultured cells were treated with BLZ945, VCR or BLZ945/VCR. Cell viability of EL4 cells after treated with different drugs were examined by CCK8 assay (right panel). (n = 3) B, Scheme of macrophage‐conditioned media (CM) preparation and apoptosis analysis of EL4 cells. EL4 cells were cultured for 48 h in order to collect EL4‐CM. Three experimental conditions were subsequently established to produce BMDM conditioned media (48 h): media supplemented with M‐CSF (BMDM‐CM), EL4‐CM (LAM‐CM) and EL4‐CM + BLZ945 (LAM‐CM + BLZ945). CM from each of these three treatment groups was then added to EL4 cells treated by VCR. C, Apoptosis of EL4 cells after treated with different macrophage CM in the presence or absence BLZ945. D, Frequency of apoptotic EL4 cells (n = 3). E, Western blots showing p‐STAT3 levels in EL4 cells treated with different conditioned media

Depletion of TAMs has been shown to enhance anti‐tumour immunity. Therefore, we evaluated the inhibitory efficiency of BLZ945 together with VCR, a standard chemotherapeutic agent used in ALL. First, we co‐cultured the EL4 cells with BMDMs in vitro and then treated with BLZ945 (100 nmol/L), VCR (75 ng/mL) or BLZ945 combine VCR (Figure 4A) and monitored the cell viability using Cell Counting Kit‐8 assay. Meanwhile, we also investigated the viability of BMDMs or EL4 cells cultured alone when treated by VCR. As shown in Figure 4A, a single treatment of BLZ945 or VCR decreased EL4 cell viability, which seemed inconsistent with the data in Figure 3A. Moreover, VCR alone significantly inhibited the cell viability of EL4 cells, but had no effects on the viability of BMDMs (Figure S3). Therefore, these findings indicated that the suppression of EL4 cell induced by BLZ945 appeared to be the consequence of an inhibition of LAMs. Combination treatment with BLZ945 and VCR resulted in significantly inhibited cell viability when compared with single treatment. Therefore, our data demonstrated that BLZ945 enhanced the anti‐tumour role of VCR and the combined treatment can efficiently inhibit cell proliferation in vitro.

As BLZ945 blocked EL4 cells from inducing LAMs polarization, we examined whether EL4 cells viability was modulated by exposure to macrophages in cell culture, and if it was abrogated by BLZ945. BMDMs were stimulated with EL4‐CM to mimic the leukaemia microenvironment (referred to “LAMs”) and subsequently added to EL4 cells in co‐culture (as shown in Figure 4B). Cell apoptotic analysis showed that EL4 cells co‐cultured with LAMs were resistant to VCR‐induced apoptosis. Interestingly, these anti‐apoptotic effects were abrogated by the addition of BLZ945 (Figure 4C,D), suggesting the LAMs dependence of this effect. Because previously data showed the production of IL‐10 was significantly increased in EL4‐CM‐treated BMDMs, we intended to study whether IL‐10 influenced the activation of the known signalling pathway of proliferation or survival within EL4 cells when co‐cultured with BMDMs, including STAT3. Levels of phosphorylated STAT3 (pSTAT3) in EL4 cells increased in response to CM from the LAMs compared with BMDM‐CM. This induction was significantly reduced when conditioned media were collected in the presence of BLZ945 (Figure 4E). Collectively, these data demonstrated that EL4 cells and macrophages had reciprocal effects on the survival, proliferation or polarization of each other to promote tumorigenesis and that this signalling pathway can be blocked by CSF‐1R inhibition.

### 3.5 Combination treatment with CSF1R inhibitor and VCR reduced T‐ALL burden and improved survival

**Figure 5:**
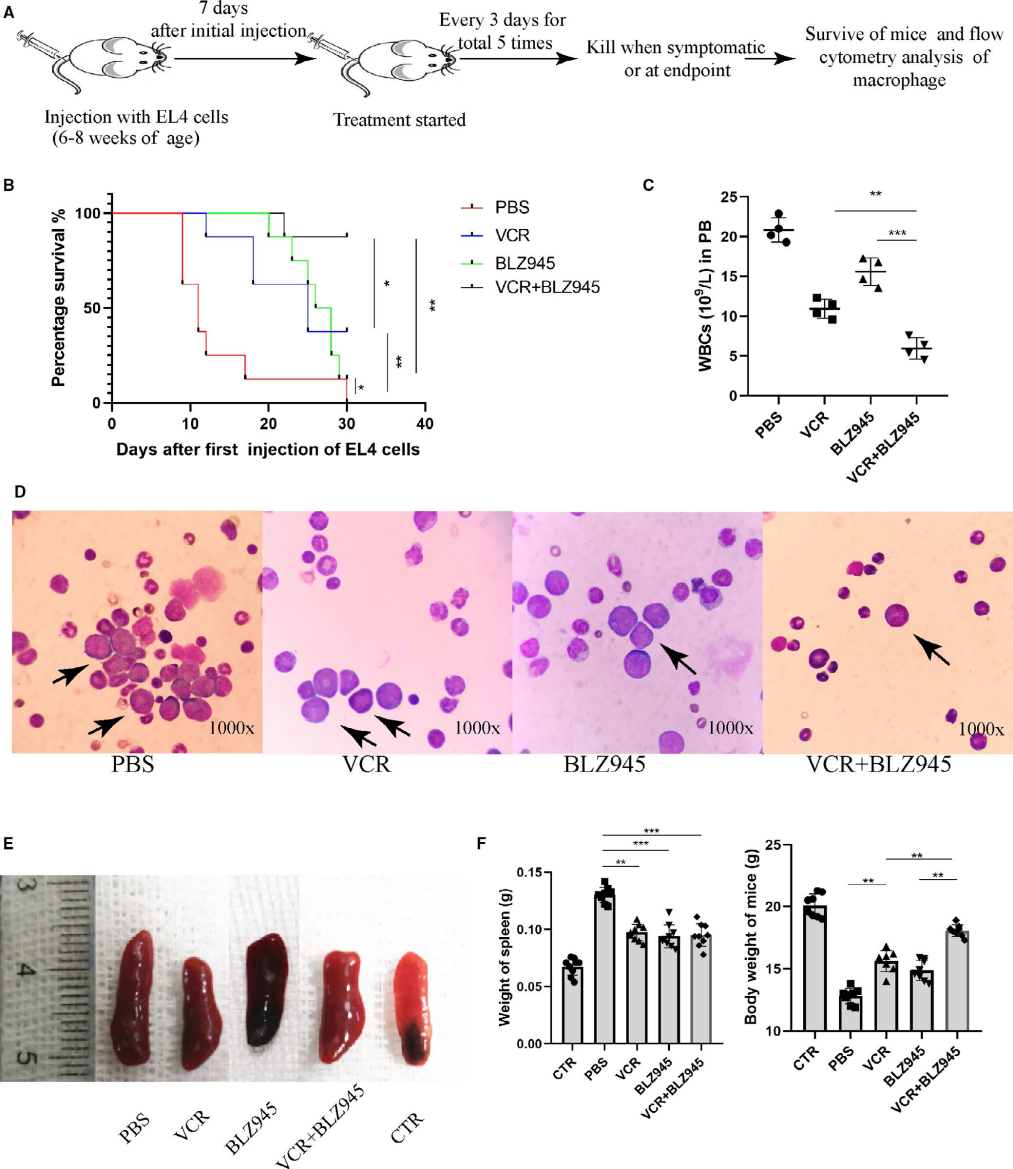
Combination therapy significantly reduces ALL burden and improves survival. A, Schematic illustration of the experimental design. Mice were killed when they developed symptoms or reached the trial endpoint and further analysis was done. B, Kaplan‐Meier survival curve of the mice in ALL models (n = 8). C, Total white blood cells count (WBC) in the peripheral blood of mice when symptomatic or at endpoint of therapy (n = 4). D, Wright staining of bone marrow smears from 3 groups of mice. Black arrow indicates blasts. E, Representative images of spleens collected from each of the different treatment groups. F, Bar graph shows the weight of spleen and body from each of the different treatment groups (n = 8)

Encouraged by the in vitro promising results of BLZ945, we decided to evaluate their in vivo anti‐tumour activities in a murine T‐ALL model. As BLZ945 treatment had no direct effects on the viability of EL4 cells but enhanced the anti‐tumour role of VCR in vitro, we intended to study the synergistic effects of BLZ945 and VCR in T‐ALL mice rather than the single effect of BLZ945 (Figure 5A). As shown in Figure 5B, mice that received VCR/BLZ945 combination treatment survived longer and had a significantly lower white blood cell (WBC) count in the PB (Figure 5C) and reduced numbers of leukaemic blast cells in the BM (Figure 5D) as compared with the PBS group or mice receiving VCR or BLZ945 monotherapy. Furthermore, splenomegaly had been observed among the T‐ALL mice, and the weight of the spleen of the combination‐treated mice decreased significantly than in the PBS group, while there was no difference when compared with VCR or BLZ945 group (Figure 5E,F). However, the bodyweight of the combination‐treated mice increased significantly than other groups (Figure 5F). Collectively, these data established that the combination of BLZ945 and VCR significantly delayed leukaemia progression and enhanced the effects of traditional chemotherapeutic drugs.

### 3.6 VCR and BLZ945 combination therapy significantly reduced the abundance of LAMs

**Figure 6:**
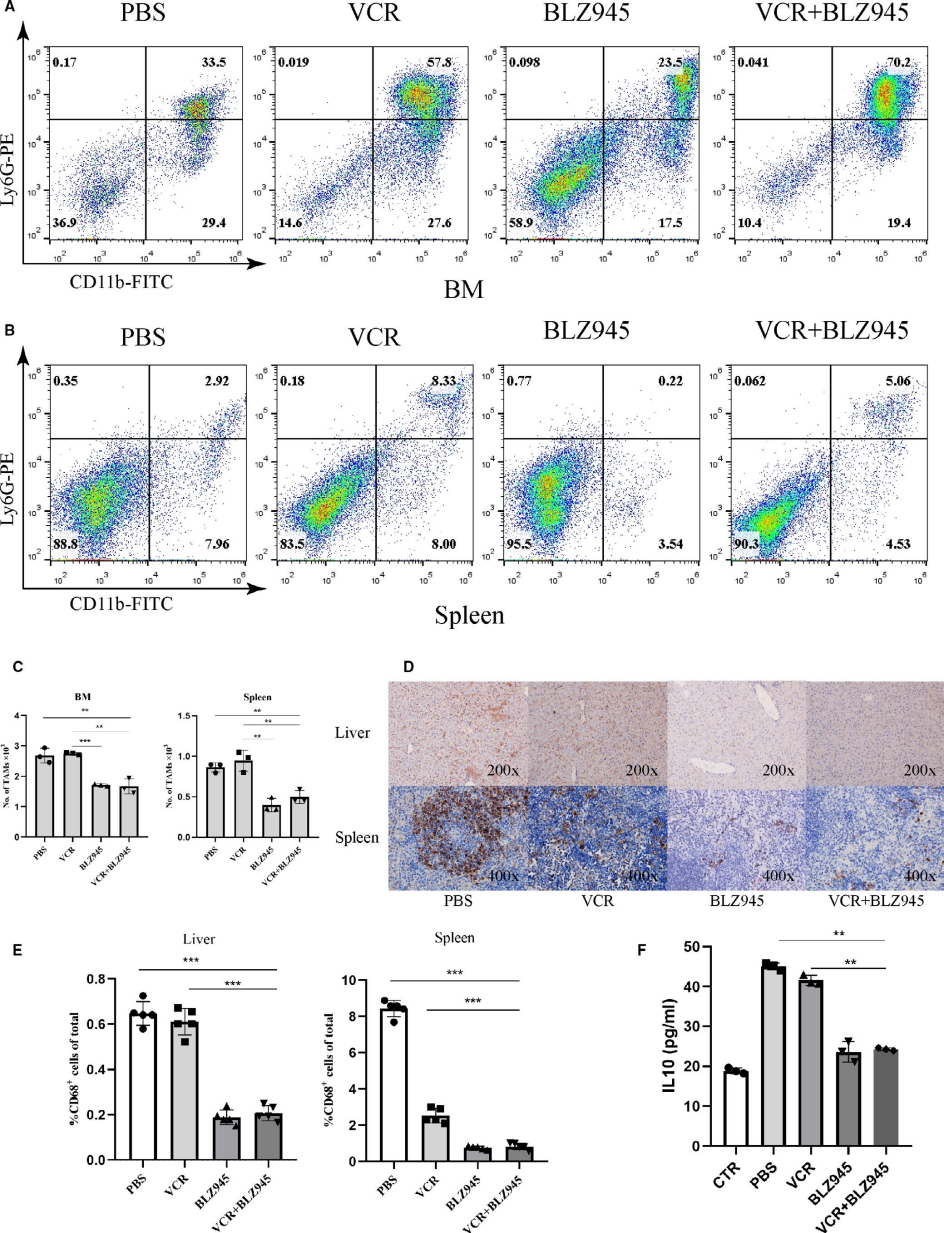
Combination therapy significantly reduces the abundance of LAMs. A, Representative FACS plots showing the ratio of CD11b^+^Ly6G^−^ macrophages in the BM derived from different treatment groups. B, Representative FACS plots showing the ratio of CD11b^+^Ly6G^−^ macrophages in the spleen derived from different treatment groups. C, Absolute numbers of CD11b^+^Ly6G^−^ macrophages in the BM (left) or spleen (right) from different treatment groups (out of 10^4^ cells acquired by FACS), (n = 3). D, Liver and spleen sections of different treatment groups were stained using an anti‐CD68 antibody to identify macrophages. E, Frequency of CD68^+^ macrophages in the liver (left) or spleen (right) of different treatment groups (n = 5). F, Level of IL‐10 in serum of mice from different treatment groups (n = 3)

Inhibition of CSF‐1R was able to deplete TAMs and inhibit their tumour‐promoting function. As depicted in Figure 6, it was clearly found that VCR monotherapy did not reduce the ratio of macrophages in the BM (Figure 6A) and spleen (Figure 6B) compared with the PBS group at the end of treatment. By contrast, combination treatment displayed a significantly reduced LAMs infiltration when compared with VCR single treatment (Figure 6A‐C). The therapeutic effect of BLZ945 on the infiltration of macrophages in the liver and spleen of tumour‐bearing mice after different treatments was further examined by IHC using an anti‐CD68 antibody. We detected decreased CD68^+^ macrophages in both the liver and spleen in VCR/BLZ945 combination‐treated mice when compared with the PBS group or VCR single‐treated mice (Figure 6D,E), indicating that VCR/BLZ945 treatment significantly reduced the abundance of macrophages in tumour‐bearing mice. Intriguingly, VCR monotherapy did not affect the ratio of CD68^+^ macrophages in the liver, whereas it significantly decreased the fraction of CD68^+^ macrophages in the spleen of T‐ALL mice. More importantly, the levels of serum IL‐10 in T‐ALL and VCR‐treated mice was significantly increased than in the normal mice, which significantly decreased in BLZ945 and VCR/BLZ945 treated group (Figure 6F), consistent with previously results in vitro.

## 4 DISCUSSION

TAMs are known to play a crucial role in tumour development, invasion and metastases. Increasing evidence suggests TAMs have been correlated with a poor prognosis in different types of cancers including prostate, gliomas, ovarian and cervical carcinomas.^18^, ^26^, ^27^, ^28^ TAMs are a potential target for developing novel therapeutic strategies in solid tumours. However, little is known about the molecular interplay between leukaemia cells and the associated macrophages.

TAMs from the BM and spleen in the T‐ALL mouse showed considerable diversity in functions and gene expression profiles.^29^ TAMs have also been isolated from the PB, spleen and lymph nodes in CLL patients and they have been shown to be essential for CLL cell survival in the tumour microenvironment.^19^, ^23^ In this setting, these TAMs, also known as nurse‐like cells, share a similar gene expression profile to TAMs derived from other tumour types. The frequency of CD163^+^CD206^+^ M2‐like macrophages in the BM of AML patients was significantly elevated compared with healthy volunteers. Moreover, more M2‐like macrophages correlated with a worse prognosis in AML patients.^21^ In the present study, we observed an increased accumulation of CD11b^+^Ly6G^−^ macrophages in the BM and spleen of T‐ALL mouse models that is consistent with previous reports.^15^ Macrophages infiltration was further verified in the liver and spleen by CD68 IHC.

TAMs are recognized as M2‐like macrophages, which is associated with pro‐tumoural functions. As previously reported, increased expression of M2‐associated genes was detected in cultured BMDMs when exposed to glioma cell‐conditioned media and that was blocked in the presence of CSF‐1R inhibitor.^18^ Mantle cell lymphoma (MCL)–associated macrophages (MϕMCL) shared more similarities with M2 macrophages.^20^ Further, using the small‐molecule GW2580 efficiently counteracted MϕMCL pro‐tumoural effects ex vivo. However, MϕMCL expressed not only M2‐associated soluble factors but also M1‐associated soluble factors. This suggested the phenotypic and functional diversity of macrophage polarization in MCL. To investigate the effect of T‐ALL cells on the macrophage phenotypes, we first co‐cultured BMDMs with EL4 cells in vitro. We observed that lymphoblastic EL4 cells can polarize BMDMs into LAMs with M2‐like characteristics in vitro and that this can be blocked by BLZ945, a CSF‐1R inhibitor.

Next, we further studied the effect of BLZ945 on the mutual interaction of LAMs and leukaemia cells. Our data demonstrated that BLZ945/VCR treatment significantly inhibited EL4 cells proliferation compared with single treatment, indicating that BLZ945 could improve the efficacy of VCR chemotherapy in vitro. Next, we found that EL4 cells were significantly resistant to apoptosis treated by VCR when co‐cultured with LAMs, and this was abrogated by BLZ945. Given the importance of soluble factors on protecting leukaemia cells from apoptosis in the microenvironment, we next sought to investigate one of the possible cytokines secreted by EL4‐treated BMDMs that may be involved in the maintenance of EL4 cells survival. We focused our attention on IL‐10 as a survival factor that may be involved in this effect. IL‐10, an immuno‐regulatory cytokine, is well known for its immunosuppressive function and contributes to the maintenance of an immunosuppressive milieu within the tumour environment. IL‐10 enhances the survival of CLL cells by acting as an autocrine growth factor and it is able to reduce cell death caused by hydrocortisone. In addition, a significantly higher level of IL‐10 was detected in the plasma of MCL patients compared with age‐matched healthy donors. As showed above, IL‐10 mRNA as well as its supernatant concentration was significantly increased in BMDMs when co‐cultured with EL4‐CM, and this can be blocked by BLZ945. Furthermore, STAT3 activation was found in EL4 cells when treated with LAM‐CM, and this role was blocked in the presence of BLZ945. STAT3 activation is a common feature of tumour cells within the tumour stroma, and it is thought to prevent cell apoptosis and enhance tumour cell growth and metastasis.^30^, ^31^ Collectively, these data indicate that leukaemia cells polarize macrophages into alternatively activated M2‐like LAMs which in turn protects leukaemia cell from apoptosis. The signalling between macrophages and leukaemia cells can be abrogated by CSF‐1R inhibition.

CSF‐1 and its receptor, CSF‐1R, are viewed as the primary signalling pathways for functional maintenance of TAMs. Inhibition of CSF‐1R was able to deplete TAMs and inhibit their tumour‐promoting function. A recent study reported that CSF‐1R inhibitors exhibited anti‐tumour activity in AML by blocking paracrine signals from support cells.^21^ Moreover, the effect of CSF‐1R inhibitors has been also shown in multiple myeloma and in CLL.^19^, ^22^ BLZ945 is a CSF‐1R inhibitor that selectively binds to CSF‐1R expressed on TAMs and inhibits the proliferation of TAMs.^32^, ^33^, ^34^ In our study, we found that the combination of VCR and BLZ945 blocked T‐ALL progression and significantly improved survival, supporting that BLZ945 can enhance the anti‐tumour role of VCR. Furthermore, research involved CLL indicates that CLL cell growth in vivo is critically dependent on the support of TAMs and show the tangible positive effects of manipulating such support.^19^ Targeting monocytes/macrophages by CSF1R inhibition can be exploited as a therapeutic strategy in CLL. Macrophage depletion significantly reduced the number of leukaemic B cells in the BM of CLL mice. CSF1R blockade was able to stabilize the disease and induce increasing necrosis of the splenic leukaemic cells over time. This anti‐leukaemic effect was associated with a remarkable and selective depletion of CSF1R^+^ MRC1^+^ M2‐like TAMs.^19^ However, there was no marked difference in the size of the spleen between VCR and VCR/BLZ945, a finding that requires further research. Most importantly, combination therapy significantly reduced the abundance of macrophages in tumour‐bearing mice and promoted the anti‐tumour activity of VCR. It is noticeable that the frequency of macrophages in the spleen between the PBS and VCR group is inconsistent as detected by IHC and flow cytometry analysis. The reason for this may be that we identified macrophages in the spleen by IHC using CD68. CD68 is a receptor expressed by tissue macrophages and generally considered to be a pan‐macrophage marker, which cannot distinguish M1 or M2 subtypes from all the infiltrated macrophages.^24^ What's more, although CD68 is a well‐accepted marker of macrophages, other cell types also express CD68, including mast cells, endothelial cells, fibroblasts and osteoclasts.^35^, ^36^, ^37^ Because we were limited to one marker for macrophage identification, the other possible cell types expressing CD68 were not ruled out. As is well known, VCR as a chemotherapy drug not only inhibits proliferation and survival of tumour cells but is also toxic to many healthy cell types.^38^, ^39^ Therefore, we suspect that VCR perhaps inhibits other CD68‐expressing cells rather than macrophages in the spleen of T‐ALL mice, which needs further investigation. What's more, the serum IL‐10 concentration was also increased in leukaemia mice and decreased when treated by VCR/BLZ945.

In 2015, Chen et al studied the dynamic distribution, dynamic expression of phenotype‐associated genes and function of LAMs in a Notch1‐induced mouse model of T‐ALL.^29^ They found that the characteristics of BM and spleen LAMs had considerable differences, which might play an important role in the pathological process of T‐ALL. Moreover, LAMs could be subdivided into M1‐like (CD206^−^) and M2‐like (CD206^+^) groups expressed M1‐ or M2‐associated genes. These results suggested the functional and phenotypic characteristics of LAMs (M1‐like or M2‐like), which were modified by organ specific microenvironments.

In addition, research indicates that AML leads to the invasion of AML‐associated macrophages into the BM and spleen of leukaemic mice.^15^ In different leukaemic mouse models, these macrophages support the in vitro expansion of AML‐cell lines better than macrophages from non‐leukaemic mice. Moreover, they also found that the transcriptional repressor growth factor independence 1 (Gfi1) is crucial in the process of macrophage polarization, since its absence impedes macrophage polarization towards a leukaemia‐supporting state and favours an anti‐tumour state both in vitro and in vivo. These results not only suggest that AML‐associated macrophages play an important role in the progression of acute myeloid leukaemia, but also implicate Gfi1 as a pivotal factor in macrophage polarization.

In summary, we demonstrated that LAMs accumulated in the BM and spleen of T‐ALL mice. Depletion of LAMs by a CSF‐1R inhibitor enhanced the anti‐tumour effects of VCR in mice with T‐ALL. Our study shed new light on the importance of the microenvironment in T‐ALL and showed that macrophages are a potential target for developing novel therapeutic strategies in T‐ALL.

## CONFLICT OF INTEREST

None.

## AUTHOR CONTRIBUTION

**Kun Li:** Investigation (lead); Methodology (lead); Project administration (lead); Writing‐original draft (lead). **Wenfu Xu:** Methodology (supporting); Project administration (supporting). **Ke Lu:** Methodology (supporting); Project administration (supporting). **Yuxi Wen:** Investigation (supporting); Project administration (supporting). **Tianqing Xin:** Investigation (supporting). **Yaqing Shen:** Project administration (supporting); Resources (supporting). **Xueyan Lv:** Methodology (supporting). **Shimin Hu:** Formal analysis (supporting); Writing‐review & editing (supporting). **Run‐Ming Jin:** Conceptualization (supporting); Funding acquisition (lead); Validation (supporting); Writing‐review & editing (supporting). **Xiaoyan Wu:** Conceptualization (lead); Funding acquisition (lead); Validation (supporting).

## Supporting information

Supplementary Material
