# Supplementary material for: CSF‐1R inhibition disrupts the dialog between leukaemia cells and macrophages and delays leukaemia progression
Source: J Cell Mol Med. 2020 Oct 10;24(22):13115–28. doi: 10.1111/jcmm.15916 (PMC7701573; doi:10.1111/jcmm.15916)
Supplement: Supplementary file 1 — Supplementary Material [file JCMM-24-13115-s001.docx]

**CSF-1R inhibition disrupts the dialog between leukemia cells**

**and macrophages and** **delays leukemia progression**

Kun Li^1^, Wenfu Xu^1^, Ke Lu^1^, Yuxi Wen^1^, Tianqing Xin^1^, Yaqing Shen^1^, Xueyan Lv^1^, Shimin Hu^2^, Runming Jin^1*^, and Xiaoyan Wu^1*^

^1^Department of Pediatrics, Union Hospital, Tongji Medical College, Huazhong University of Science and Technology, Wuhan, China.

^2^Department of Hematopathology, The University of Texas MD Anderson Cancer Center, Houston, TX.

**Corresponding authors:**

**Xiaoyan Wu**

Department of Pediatrics, Union Hospital, Tongji Medical College, Huazhong University of Science and Technology, No. 1277 JieFang Road, Wuhan, Hubei 430022, China. E-mail: [xwu@hust.edu.cn](mailto:xwu@hust.edu.cn); [1994XH0558@hust.edu.cn](mailto:1994XH0558@hust.edu.cn). Phone: 027-85726718.

**Runming Jin**

Department of Pediatrics, Union Hospital, Tongji Medical College, Huazhong University of Science and Technology, No. 1277 JieFang Road, Wuhan, Hubei 430022, China. E-mail: [jinrunm@qq.com](mailto:jinrunm@qq.com)**.** Phone: 027-85726718.

**Additional Supporting Information**

**Table S1 Sequence of primers in this study.**

| Primers | Sequence |
| --- | --- |
| TNF-α | Forward: 5′-CAGCCTCTTCTCATTCCTGCT-3′ |
|  | Reverse: 5′-GGGTCTGGGCCATAGAACTG-3′ |
| Arg1 | Forward: 5′- CATCCAGTTGCCTTCTTGGG-3′ |
|  | Reverse: 5′- TCTTCCATCACCTTGCCAATC-3′ |
| IL-10 | Forward: 5′- TACAGCCGGGAAGACAATAACT-3′ |
|  | Reverse: 5′- AGGAGTCGGTTAGCAGTATGTTG-3′ |
| CD206 | Forward: 5′-CGTTTCGGTGGACTGTGGA-3′ |
|  | Reverse: 5′- GTTGTGGGCTCTGGTGGG-3′ |
| GAPDH | Forward: 5′- TGAAGGGTGGAGCCAAAAG-3′ |
|  | Reverse: 5′- AGTCTTCTGGGTGGCAGTGAT-3′ |

**
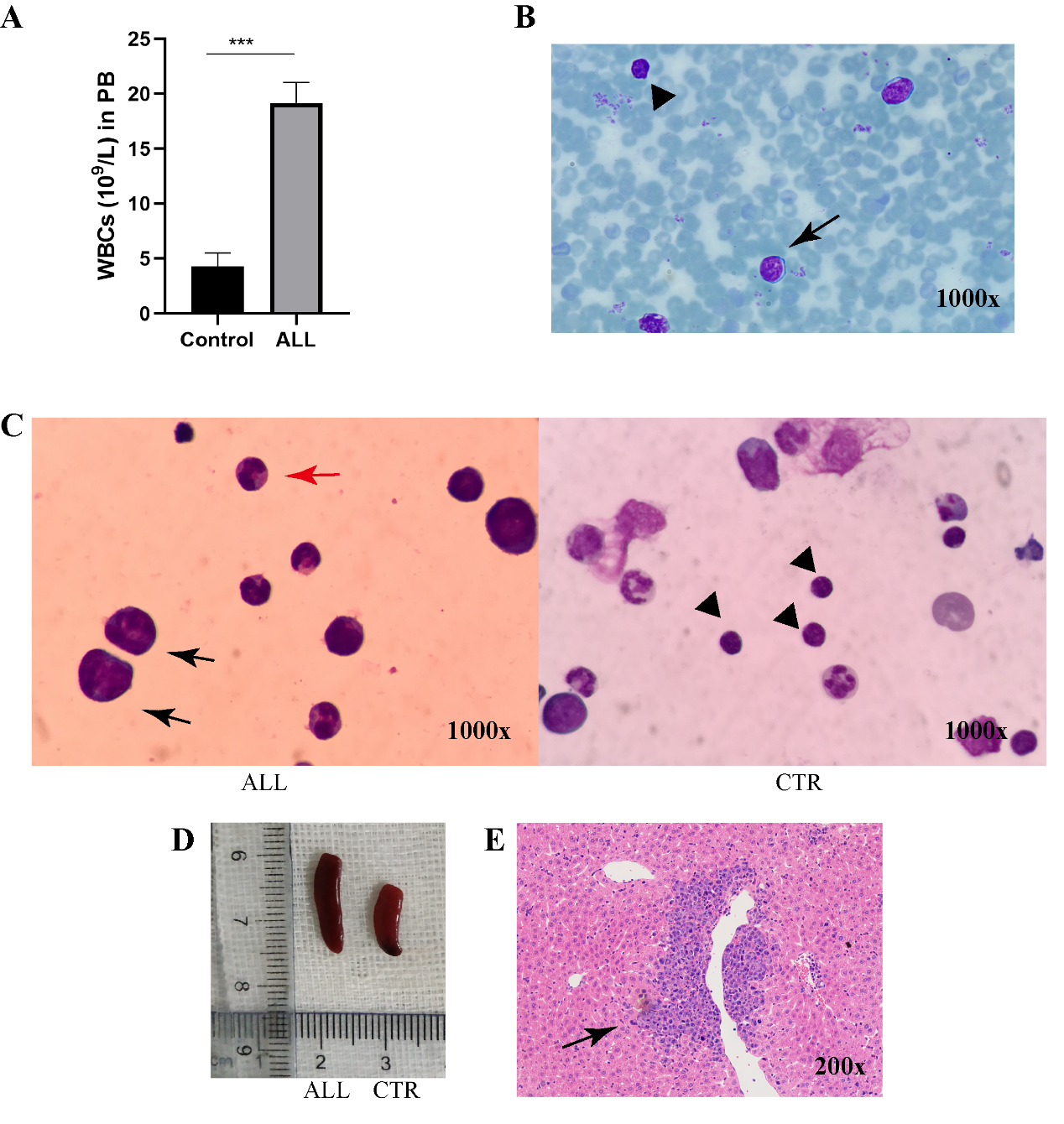
**

**Figure S1 Induction of T-ALL in C57BL/6 mice injected with the EL4 leukemia cells. (A)**Total white blood cells count (WBC) in peripheral blood of leukemic and normal mice. (B) Wright staining of blood smears of leukemic mice. Black arrow indicates blasts and arrowhead indicates lymphocyte. (C) Wright staining of bone marrow smears of leukemic (left) and normal (right) mice. Black arrows indicate blasts, red arrows indicate normal neutrophils, and arrowheads indicate lymphocyte. (D) Representative images of spleens from leukemic and normal mice. (E) H&E staining of liver sections of ALL mice. Arrows indicate blast infiltrates.


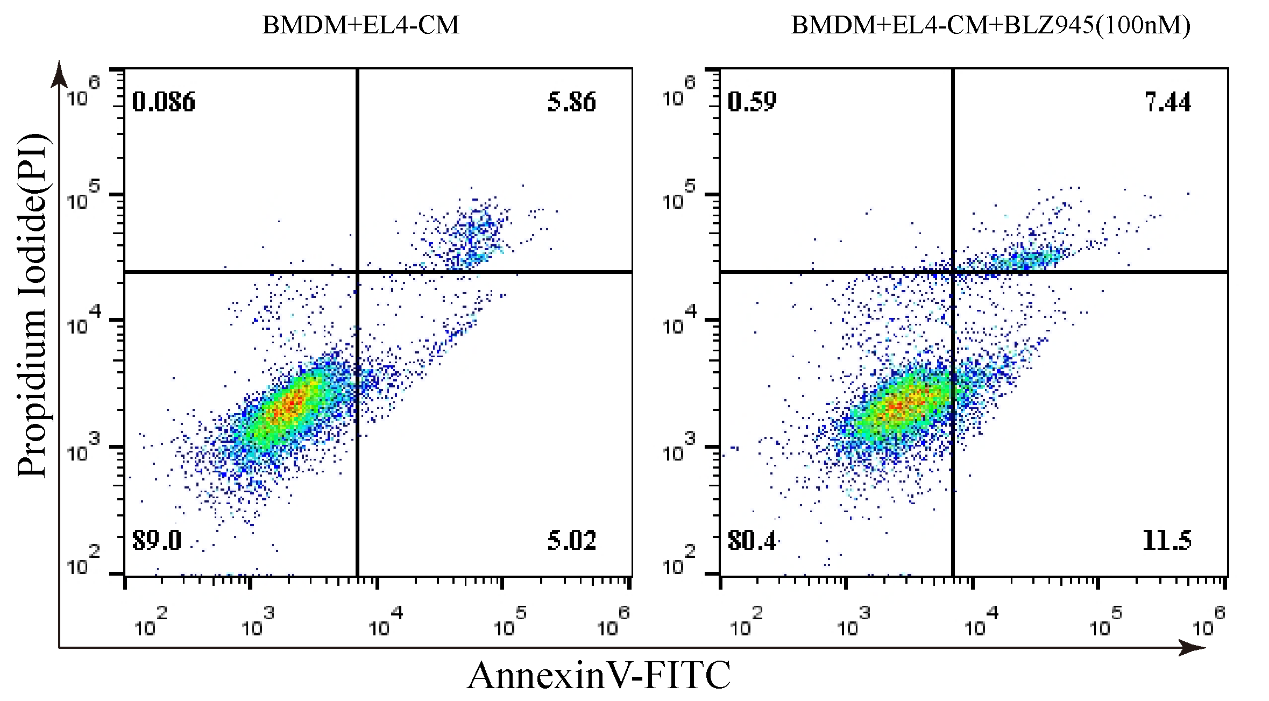


**Figure S2** **BLZ945 induce apoptosis in BMDMs** **cocultured with EL4 cells.** Apoptosis of BMDMs cocultured with EL4 cells after treated with BLZ945 (100nM) for 48 hours. Apoptotic BMDMs were detected by double staining with Annexin-V and propidium iodide, and measured by flow cytometry.


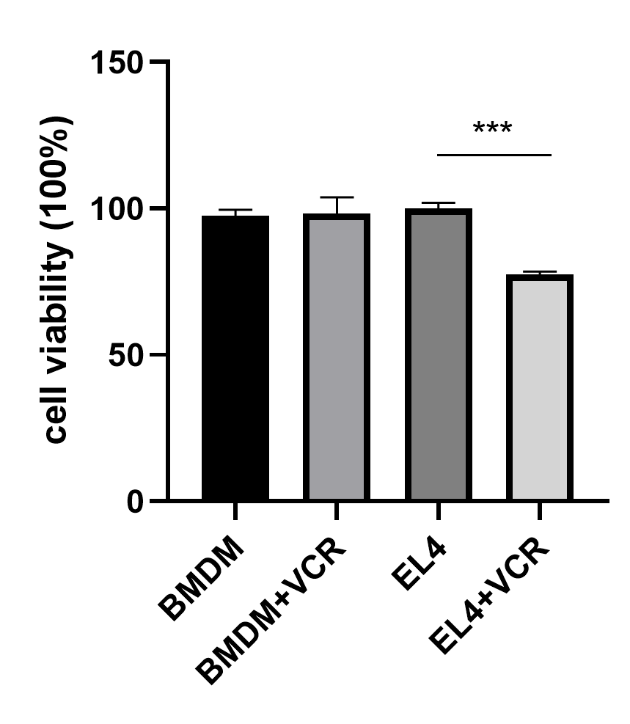


**Figure S3 Viability of BMDMs or EL4 cells cultured alone treated by VCR.** Cell viability of BMDMs or EL4 cells after treated with VCR (75ng/ml) for 48 hours (n=4).
